# The role of Poly-ADP ribose polymerase (PARP) enzymes in chemotherapy-induced cognitive impairments – parallels with other neurodegenerative disorders

**DOI:** 10.3389/fphar.2025.1615843

**Published:** 2025-06-09

**Authors:** Dahlia A. Ordaz, Kalpna Gupta, Daniela A. Bota

**Affiliations:** ^1^ Department of Pathology and Laboratory Medicine, University of California Irvine, Irvine, CA, United States; ^2^ Department of Medicine, University of California Irvine, Irvine, CA, United States; ^3^ Department of Neurological Surgery, University of California Irvine, Irvine, CA, United States; ^4^ Chao Family Comprehensive Cancer Center, University of California Irvine, Irvine, CA, United States; ^5^ Department of Neurology, University of California Irvine, Irvine, CA, United States

**Keywords:** PARP (poly(ADP-ribose), PARP inhibitors (PARPi), chemo brain, neuroinflamamation, chemotherapy

## Abstract

Poly (ADP-ribose) polymerase (PARP) enzymes are critical in repairing DNA damage induced by chemotherapy and/or radiation. Due to PARP’s role in DNA repair, inhibiting PARP leads to genomic instability and accumulation of damaged cells in cell cycle arrest. Previous studies have shown that PARP1 activation contributes to the development of various malignant disorders, and using PARP inhibitors is a promising intervention in these diseases. However, PARP activation is also common in neurological and inflammatory disorders. PARP inhibitors were studied in preclinical models of neurodegenerative disorders such as Parkinson’s, Huntington’s, and Alzheimer’s Disease (AD). In neurodegenerative disorders like AD, activated PARP1 induces Aβ and forms Tau tangles, worsening cognitive symptoms. PARP inhibitors are currently used in combination therapy with chemotherapy drugs, including cisplatin and temozolomide, which are all described as having significant rates of central and peripheral nervous system side-effects, raising the potential question of using PARP inhibition not only as a cancer treatment but as an approach to mitigate the toxicity of the cancer drugs. This review will summarize evidence for the potential use of PARP inhibitors for neurologic disorders and discuss future prospects of how PARP inhibitors could be repurposed as neuroprotective agents against the cognitive complications of chemotherapeutic drugs.

## Introduction

Chemotherapy has been the gold standard of care in cancer treatment for decades due to its ability to induce DNA damage in malignant cells. Patients can experience various nervous system complications caused by cancer and its treatments, including chemotherapeutic agents, leading to cognitive impairments, peripheral neuropathy, loss of fine motor skills, and several other symptoms. Chemotherapy-Induced Cognitive Deficits (CICD, chemo brain) is a term that refers to the long-term cognitive impairments induced by chemotherapeutics. With the recent positive trends in patient survival, there is a critical need for effective CICD treatments. Previous CICD therapies have had limited success in the significant alleviation of cognitive impairments due to the complex and poorly understood molecular mechanisms and various confounding variables. Studies have shown that Poly ADP-ribose polymerase (PARP) inhibitors are potential neuronal protectors in multiple pathologies, including neurodegenerative disorders (ND). PARP plays a critical role in repairing that damage, and PARP-1 activation contributes to the pathology of various NDs. PARP inhibitors (PARPi) are a promising targeted therapy for BRCA-mutated cancer patients, while being used in combination with chemotherapy drugs like cisplatin and temozolomide, which cause substantial amounts of central and peripheral nervous system side effects. There are many mechanistic commonalities between CICD and various NDs, including Parkinson’s, Huntington’s, and Alzheimer’s Disease (Table 1). Like many ND, chemotherapy induces reactive oxygen species (ROS) and neuroinflammation. Previous studies have documented that PARPi can prevent cell death induced by oxidative stress and counter-inflammation (Hocsak et al., 2017). Therefore, we propose the use of PARPi not only as a neuroprotective therapy for ND but also as an intervention against the neurotoxicity induced by cancer drugs.

**TABLE 1:** Comparative Features of Neurodegenerative Disorders and Cancer. This table illustrates some of the key features of neurodegenerative disorders, such as Parkinson’s, Huntington’s, and Alzheimer’s, in comparison to Cancer. This table was created using BioRender.com (Kam et al., 2018; Jarosińska and Rüdiger, 2021; McColgan and Tabrizi, 2018; Kumar and Ratan, 2016; Mehrabi et al., 2016; Kumar et al., 2023; Rajmohan and Reddy, 2017; Gulisano et al., 2018; Moreira et al., 2022; Zhang et al., 2019; Picca et al., 2021; Haga et al., 2019; Kam et al., 2020; Chen et al., 2022; Genovese et al., 2022; Wang et al., 2023; Kim et al., 2022; Ferrazzoli et al., 2016; Lovell and Markesbery, 2007; Glaviano et al., 2024; Liberti and Locasale, 2016; Quail and Joyce, 2013).

Features	Parkinson's	Huntington's	Alzheimer's	Cancer
Cancer	Depletion of dopaminergic neurons in the substantia nigra (Kam et al., 2018, Zhang et al., 2019)	Excessive repeats of CAG in the HTT gene mutant (mHTT) lead to protein aggregation (Jarosińska and Rüdiger, 2021, Kumar and Ratan, 2016)	An accumulation of β-amyloid plaques and tau tangles leads to neuronal death (Rajmohan and Reddy, 2017)	Uncontrolled cell division as a result of genetic mutations and disrupted cell cycle regulation (Glaviano et al., 2024)
Pathology	Mitochondrial dysfunction, neuroinflammation, Lewy Bodies (α-synuclein aggregation) (Kam et al., 2018, Picca et al., 2021)	Transcriptional dysregulation, mitochondrial damage, excitotoxicity, mutant huntingtin aggregates (Kumar and Ratan, 2016)	Synaptic dysfunction, neuro-inflammation, tau tangles, β-amyloid plaques (Rajmohan and Reddy, 2017, Gulisano et al., 2018)	Loss of tumor suppressor, DNA mutations, evasion of apoptosis, oncogene activation (Wang et al., 2023)
Apoptosis	Programmed cell death of dopaminergic neurons (Picca et al., 2021)	Excitotoxicity and mitochondrial failure leas to neuronal apoptosis (McColgan and Tabrizi, 2018)	Amyloid toxicity leads to neuronal apoptosis (Rajmohan and Reddy, 2017)	Cancer cells evade apoptosis through mutations like p53 and others (Genovese et al., 2022)
Protein Misfolding & Aggregation	Formation of toxic oligomers from misfolded α-synuclein forms (Picca et al., 2021)	mHTT aggregates cause impairments in cellular processes (Jarosińska and Rüdiger, 2021, Mehrabi et al., 2016)	Hyperphosphorylated tau and misfolded β-amyloid disrupt cellular function (Gulisano et al., 2018, Moreira et al., 2022)	Proteinopathies in particular cancers; otherwise not typical (Wang et al., 2023)
Mitochondrial Dysfunction	Impaired ATP production, mitochondrial DNA damage, increased oxidative stress (Haga et al., 2019)	Altered energy metabolism and mitochondrial fragmentation (Kumar and Ratan, 2016)	Neurodegeneration as a result of oxidative stress (Glaviano et al., 2024)	Warburg Effect- certain types of cancers are dependent on an altered metabolism (Liberti and Locasale, 2016)
Cellular Impact	Degeneration of dopaminergic neuron in the basal ganglia which leads to motor deficits (Picca et al., 2021)	Neuronal loss in the striatum and cortex affects movement and cognition (Kumar and Ratan, 2016)	Extensive neuronal death, in particular the cortex and hippocampus (Zhang et al., 2019)	An invasion of surrounding tissue, uncontrolled cell division, and metastasis (Glaviano et al., 2024)
DNA Damage & Repair	Neurodegeneration can a be direct result of impaired DNA repair mechanisms (Wang et al., 2023)	mHTT contributes to the disruption of DNA repair pathways (Kumar and Ratan, 2016, Kumar and Ratan, 2016)	Oxidative DNA damage worsens disease progression (Glaviano et al., 2024)	Defects in DNA repair leads to genomic instability and DNA mutations with uncontrolled growth (Genovese et al., 2022)
Inflammation and Immune Response	Activated microglia and astrocytes induces chronic neuroinflammation (Picca et al., 2021,Kam et al., 2020)	Progression due to dysfunctional glial and immune response (McColgan and Tabrizi, 2018)	Neuroinflammation, and microglial activation (Rajmohan and Reddy, 2017)	Tumor microenvironment and inflammatory cytokines can promote or suppress tumor growth (Liberti and Locasale, 2016)
Disease Progression	Progressive loss of motor function, non-motor symptoms (ex: cognitive) (Kam et al., 2018)	Psychiatric symptoms, cognitive decline, and progressive motor dysfunction (Jarosińska and Rüdiger, 2021, McColgan and Tabrizi, 2018)	Progressive motor dysfunction, psychiatric symptoms, behavioral changes, and cognitive decline (Moreira et al., 2022)	Progression is dependent on cancer. Slow or aggressive can lead to metastasis (Quail and Joyce, 2013)
Therapeutic Targets	α-synuclein aggregation inhibitors, neuroprotective agents, and dopamine replacement (Ferrazzoli et al., 2016, Lovell and Markesbery, 2007)	Protein aggregation inhibitors, mitochondria protectors, and gene slicing therapies (Jarosińska and Rüdiger, 2021, McColgan and Tabrizi, 2018, Mehrabi et al., 2016)	Acetylcholinesterase inhibitors, neuroprotective agents, β-amyloid/ tau targeting therapies (Moreira et al., 2022)	Chemotherapy, radiation, surgery, targeted therapies (ex: immunotherapies) (Genovese et al., 2022)

### PARP and its role in cancer

The PARP enzymes are a family of 17 members, each with different roles. PARP-1 is the most notable member due to its role in oncology treatments and its first responder-like behavior in DNA damage repair. PARP-1 is a large protein comprised of three main domains: the DNA-binding domain, the catalytic domain, and the auto-modification domain. When PARP-1 becomes overactive, it disrupts the normal regulation of cellular processes such as cell division, apoptosis, and autophagy, optimizing tumor development and growth conditions. PARPi halts proliferation by destabilizing the replication fork by trapping the PARP on DNA lesions, thus preventing the repair of single-strand breaks (SSBs), which would then collide with the replication fork, causing a stall and eventual collapse, leading to double-strand breaks. PARPi has been combined with various other drugs, including anti-angiogenic, PI3K/AKT, epigenetic drugs, immune checkpoint inhibitors, and chemotherapy (Soung and Chung, 2023). Combining chemo drugs like cisplatin with PARPi is a novel cancer strategy because the two compounds can work synergistically to enhance their ability to kill cancer cells, especially in tumors with altered abilities in DNA repair mechanisms like BRCA mutations or compromised homologous recombination (HR) repair mechanisms (McQuade et al., 2018).

### Excessive PARP1 activation is associated with chronic inflammation and cancer

Research has shown that chronic inflammation is the foundation of a range of diseases, including cancer, Parkinson’s Disease (PD), Huntington’s Disease (HD), autism spectrum disorder (ASD), Alzheimer’s Disease (AD), diabetes, and cardiovascular disorders. Inflammation is one of the hallmarks of cancer, with a symbiotic relationship, meaning it can promote cancer development and be induced by the development and progression of cancer (Pazzaglia and Pioli, 2019). Interestingly, previous studies indicated that PARP-1 knockout mice were spared from inflammatory/autoimmune diseases or chronic infections involved in cancer development and progression (EZ et al., 2015).

Unfortunately, chemotherapy worsens inflammation by exacerbating the body’s natural systemic inflammatory response. PAR secreted from necrotic cells can stimulate proinflammatory signaling and increase the production of IL-6, IL-8, IL-1β, and TNF-α proinflammatory cytokines (Murata et al., 2019). In addition, excessive activation of PARP1 in microglia leads to glutamate uptake and neuronal injuries, which are associated with chronic neuroinflammation in neurological disorders (Mao and Zhang, 2022).

### Chemotherapy-induced cognitive deficits (CICD)

**FIGURE 1:**
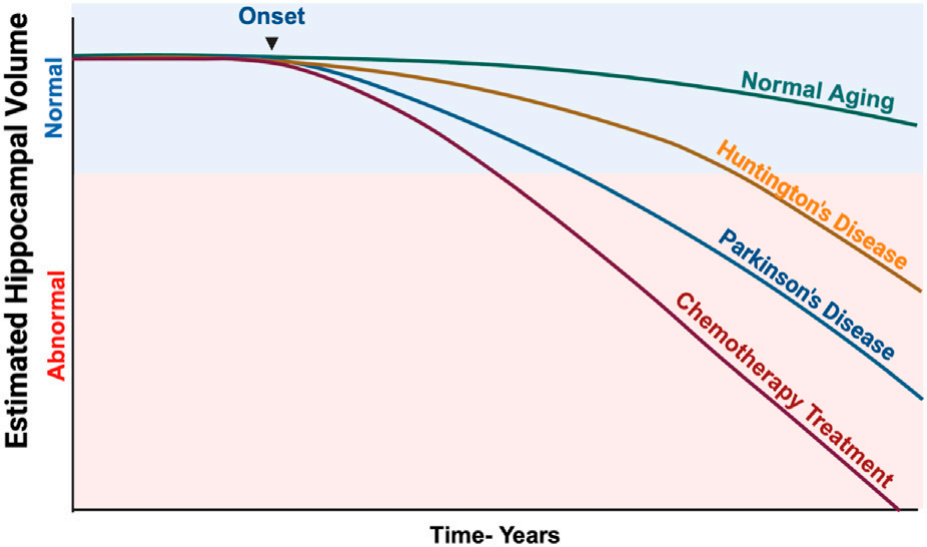
Estimated Hippocampal Volume Loss in Various Neurodegenerative Diseases and Chemotherapy-Treated Patients. This illustration shows how dramatically chemotherapeutic agents can impact the hippocampal volume, which directly correlates with significant cognitive impairments. Data was derived from several research studies that analyzed volumetric changes in the hippocampus over a prolonged period. Illustration created using BioRender.com (Pieperhoff et al., 2022; Nolen et al., 2016; Wilkes et al., 2023).

Chemotherapeutic drugs were designed to cause DNA damage in rapidly dividing cells, particularly cancer cells, but healthy cells are also affected. “Chemo-brain” or “Chemo-fog” is a term used to describe the cancer-related cognitive impairments that occur as a consequence of chemotherapeutic treatments. These cognitive impairments can affect patients for weeks, years, or lifetimes during the treatment and even after the discontinuation of chemotherapy (Figure 1). In response to DNA damage, PARP induction causes a depletion of NAD + cellular levels, leading to energy deficiency and cell death, ultimately contributing to cognitive impairments (Murata et al., 2019). An adverse side effect of chemotherapy is an inflammatory response in the brain (Schroyen et al., 2021). PARP regulates the inflammatory responses, and hyperactivation of PARP is associated with the production of pro-inflammatory cytokines by microglia and astrocytes, affecting neuronal function and contributing to cognitive deficits. Previous clinical trials have proposed pharmacotherapeutic interventions like central nervous system stimulants as a way to manage CICD but have had limited success (Karschnia et al., 2019). In the absence of effective pharmacologic treatments to mitigate CICD, the use of PARPi is hypothesized to potentially open new avenues in preventing neuronal and neural stem cell damage and the ensuing cognitive impairments.

### Sex differences in CICD

There is limited research on the sex differences on the effects of chemotherapy-induced cognitive deficits. Both men and women can experience CICD, but females are more frequently affected and studied, particularly the subset of breast cancer patients. In addition, a study conducted on long-term survivors of childhood cancer showed that females were at greater risk of developing CICD and having long-term side effects (Jacola et al., 2016).

### PARP’s role in various neurological disorders

**FIGURE 2:**
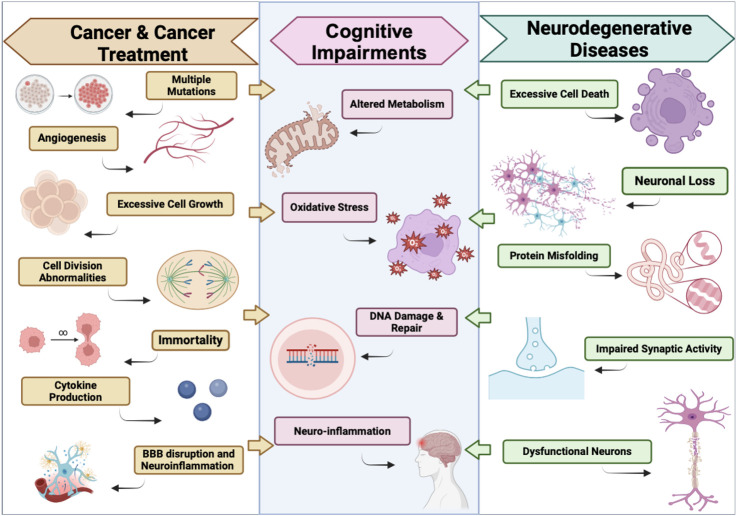
Parallels between the effects of Cancer & Cancer Treatments on the Brain and Neurodegenerative Diseases. This illustration defines some key similarities between the Pathology of Cancer & Cancer Treatments Induced Neurotoxicity, and neurodegenerative diseases that lead to the development of cognitive impairments. Illustration created using BioRender.com.

In neurological disorders like AD, PD, and HD, the common driving factors to disease progression are DNA damage, mitochondrial impairment, neuroinflammation, and oxidative stress, as represented in Figure 2 (Yan et al., 2022). Although PARP1 activation is essential in facilitating the DNA repair mechanism, excessive amounts of PARP activation exacerbate cell health and further contribute to these neurological disorders (Hu et al., 2023/11). Slightly elevated levels of PARP1 activity are initiated in response to minuscule amounts of DNA damage induced by the early stages of disease. As the disease progresses, so does extensive DNA damage, causing a cascade of predisposed factors that trigger heightened PARP1 activation. These events can induce parthanatos, or PARP1-mediated cell death, neuroinflammation, and aggravate disease pathology.

### Influence of aging on PARP effects

Age is defined as the phenomenon of adaptation to our body’s ever-changing physical and psychological environment. Aging significantly increases the risk of developing neurodegenerative diseases (Mao and Zhang, 2022) and cancer. With age comes inadequate telomerase expression, as well as telomere shortening (Kesäniemi et al., 2019). Previous studies have shown the relationship between p53 and PARP1 and their role in telomere shortening (Maresca et al., 2023). P53 is responsible for cell cycle arrest, promoting apoptotic activity, and telomere shortening (Mao and Zhang, 2022). In aging, PARP1 binding is critical for p53 transcriptional activity because it interferes with the p53 and chromosomal region maintenance one protein binding, leading to the nuclear accumulation of p53 (Ying et al., 2016). As one ages, PARP’s role becomes more integrated in the development of NDs and cancer. DNA repair is a shared hallmark of the two (Clarke and Mostoslavsky, 2022).

### Alzheimer’s disease

The pathogenesis of AD involves the accumulation of two neurotoxic protein aggregates in the central nervous system: amyloid-β (Αβ) peptide and hyperphosphorylated tau proteins (Maggiore et al., 2022). PARP-1 serves as a DNA repair enzyme, but when the cell suffers extensive DNA damage, it becomes depleted of NAD+ and ATP, which leads to parthanatos (Salech et al., 2020). Degenerating tissues and the deposition of highly insoluble materials stimulate inflammatory responses (Akiyama et al., 2000). PARP-1 overactivation is critical in the Αβ deposition and tau tangle formation. The use of PARP-1 inhibitors exerts a protective effect against neurodegeneration by diminishing neuroinflammation and microglial activation (Salech et al., 2020).

### Parkinson’s disease

PD is characterized by an accumulation of α-synuclein in Lewy bodies and Lewy neurites (Bloem et al., 2021). α-synuclein pre-formed fibrils (α-syn PFF) kill neurons by activating PARP-1 in cell death through parthanatos (Hocsak et al., 2017; Galluzzi et al., 2018). Inhibition of PARP prevents neurodegeneration and behavioral deficits caused by introducing α-syn PFF (Kam et al., 2018). α-syn PFF induces inflammatory mediator activation, which can contribute to cell death and neuroprotection by a caspase inhibitor (Codolo et al., 2013). The PARP-1 inhibitors in PD have neuroprotective effects against neurodegeneration (Outeiro et al., 2007).

### Huntington’s disease

HD is defined as a CAG nucleotide expansion correlated with length and age at onset (Author Anonymous, 1993). The excessive CAG repeats and glutamine residues lead to protein misfolding and accumulation of inclusions that trigger neuronal dysfunction and eventual neurodegeneration. These neuronal intranuclear inclusions occur because of mutated huntingtin, thus interacting and impairing several cellular functions (DiFiglia et al., 1997; Sugars and Rubinsztein, 2003). Significantly high PARP expression has been detected in neurons and glial cells in patients with HD (Vis et al., 2005). PARPi can block the formation of Poly-ADP ribose (PAR), reducing inflammation and protecting against cell death.

### Autism

ASD encompasses a range of neurodevelopmental disorders involving impairments in communication, repetitive behaviors, and social interactions. PARP-1 activity is increased in response to oxidative stress, which is often found in individuals with autism. Increased oxidative stress often leads to neuroinflammation, which is associated with autism (Sriram et al., 2015). By regulating oxidative stress responses, PARP-1 can indirectly influence the neuroinflammatory pathways in ASD. The inhibition of PARP-1 has been shown to prevent neurobehavioral and neurochemical abnormalities (Sriram et al., 2015).

A study conducted by Ahmad et al., 2020 (Ahmad et al., 2020), demonstrated that 5-AIQ, a PARPi, reduced repetitive behavior and increased social interactions, which could be indicative of the restoration of immune function. The use of PARPi has been shown to be a promising strategy for new molecular targets involved in the development of neuroimmune dysfunctions in ASD.

### Role of the other PARP members in CICD and neurodegeneration

There are various members of the PARP family, each with unique roles in cellular processes. The roles of PARP2 closely overlap with those of PARP1. PARP3 is a critical player in the repair of double-stranded breaks and genomic stability in neurons, but its role in neurodegeneration is less well known (Boehler et al., 2011). PARP6, on the other hand, is important in neuronal development, axon formation, and various mutations that are linked to neurodevelopmental disorders (Huang et al., 2016). PARP7, 9, 10, 12, and 14 have various roles in inflammatory signaling (Ke et al., 2019; Dhoonmoon and Nicolae, 2023; Welsby et al., 2014). Despite the structural similarities between the multitude of PARP family members, they differ in catalytic activity and cellular localization, which might differentially impact neuronal survival, repair, and neuroinflammation.

### The therapeutic window and timing of PARP inhibitors as an approach to preventing chemotherapy-induced cognitive disorders

PARPi are FDA-approved treatments for several types of cancer with evidence of homologous recombination deficiency (HRD). Niraparib was approved in 2017 as a maintenance therapy for platinum-based chemotherapy patients with recurrent epithelial ovarian cancer who responded positively to cisplatin (Mirza et al., 2016-12). It is typically recommended to start niraparib maintenance within 8 weeks of the last dose of the platinum chemotherapy. The current standard of care suggests that patients stay on maintenance therapy for a minimum of 36 months, unless there is disease progression or unacceptable toxicities (Lee, 2021). In a study conducted by Póti et al., it was suggested that the long-term use of a PARP inhibitor, niraparib, did not cause significant mutagenicity in cell line models and tumor xenografts (Póti et al., 2018). Despite the established benefits of PARPi, the increased use of PARPi in the clinical setting has raised awareness about PARPi resistance. These resistance mechanisms include BRCA reversion mutations, HR restoration, drug efflux increase, and the stabilization of the DNA replication fork (Giudice et al., 2022). The most common adverse events that occur with prolonged use of PARPi include gastrointestinal toxicity, hematological issues, and fatigue (Tian et al., 2022).

Several challenges present themselves in translating the use of PARPi in preclinical settings to clinical use, like optimizing dosing and timing, drug delivery to the brain, identifying predictive biomarkers, understanding resistance mechanisms, and establishing efficacy as a single agent or in combination (Vinayak and Ford, 2010). It is essential that we continue researching drug delivery and targeting strategies to maximize the therapeutic potential of PARPi while minimizing their toxicity.

## Conclusion

**FIGURE 3:**
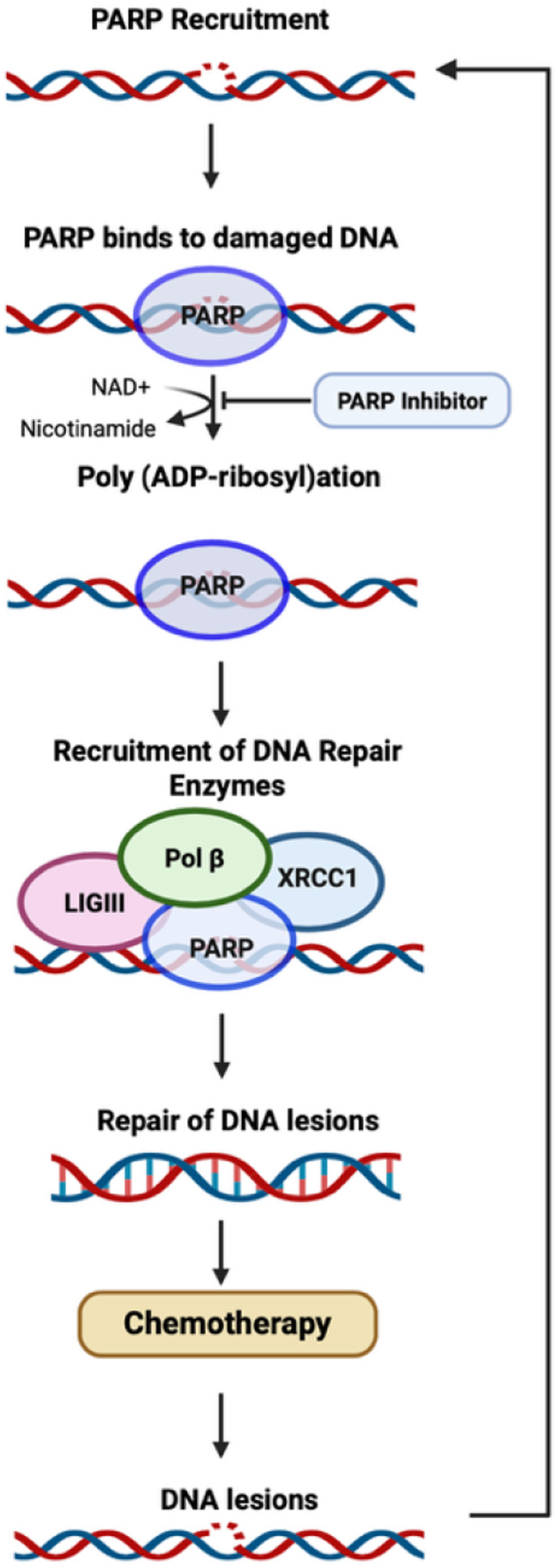
Mechanism of PARP. A schematic representation of PARP recruitment after the detection of DNA damage, and the proposed mechanism of action for PARP inhibitors (Demin et al., 2021).

PARPi have shown tremendous potential in the cancer field, with their anti-tumor effectiveness when combined with chemotherapeutic drugs (Figure 3), and tremendous potential in the treatment of various neurodegenerative disorders. This review introduces the idea of repurposing PARP inhibitors to mitigate the CICD due to the benefits already shown in preclinical models of various non-oncological neurodegenerative disorders. The neuroprotective and anti-inflammatory activity of PARPi in targeting neuronal injury in neurodegenerative conditions shows the potential of PARPi in preventing and/or mitigating CICD.
